# Inaugural Editorial - Painless Publishing in the Field of Pain

**DOI:** 10.1080/24740527.2017.1297577

**Published:** 2017-03-10

**Authors:** Joel Katz

**Affiliations:** Editor-in-Chief, Canadian Journal of Pain

It’s an honor and a pleasure to have been selected as the inaugural Editor-in-Chief of the *Canadian Journal of Pain*, the official journal of the Canadian Pain Society. The Canadian Pain Society has been without a journal for approximately one year—and it would have been much longer—but thanks to the Board of Directors of the Canadian Pain Society, and in particular to Professor Jeffrey Mogil’s tireless efforts, we’re delighted to have found a home with Taylor & Francis for the new *Journal*. It’s especially pleasing for me to be the inaugural Editor-in-Chief because of Canada’s longstanding reputation as a leader in the field, having produced so many outstanding pain scientists and clinicians over the years.

The *Canadian Journal of Pain* is a peer-reviewed, Open Access journal that publishes original articles from all disciplines involved in the study of pain, including the clinical and basic sciences, epidemiology, and health policy and health systems. The *Journal* welcomes manuscripts presenting data from original experiments, studies, or clinical trials and will also publish scholarly review articles, meta-analyses, case series and reports, commentaries, editorials, and letters to the editor. The aims of the *Journal* are to advance the understanding and treatment of pain by providing clinicians, scientists and other health care professionals with an interdisciplinary forum in which to share cutting-edge information about new developments in the field of pain.

The new Canadian Pain Society journal will give our members a high-quality outlet for their research and, at the same time, allow the *Journal* to showcase the research of our many talented members. Of course we’re “open for business” to the international community as well and we welcome manuscripts from all over the world.

There are many advantages to publishing in the *Canadian Journal of Pain*:

- The *Journal* is fully Open Access so authors retain copyright of their own work and are permitted unrestricted use, distribution, and reproduction provided the original work is properly cited;
- Taylor & Francis has waived publication fees for 2017 and has reduced the fees for 2018;
- The Editorial Board is a “who’s who” of pain, including world-renowned scientists and clinicians committed to upholding the high standards of the *Journal*;
- Experienced manuscript reviewers and receptive editorial staff who will work with authors to ensure as positive an experience as possible;
- An expeditious and streamlined review process overseen by highly experienced editorial staff to minimize time from manuscript submission to publication;
- Option to submit and publish a manuscript in French;
- Articles published in the *Journal* will have their abstracts translated into French at no cost to the authors;
- Each year, the Canadian Pain Society will publish the abstracts of its annual meeting in the *Canadian Journal of Pain* providing the international community with free and open access to the meeting’s content, and presenters with a permanent record of their poster and podium presentation abstracts.

Taken together, the *Canadian Journal of Pain* is an attractive and reliable outlet for publishing pain-related content in today’s competitive market. Moreover with the recent demise of Beall’s list of “predatory” publishers and journals, authors seeking publication will have an increasingly difficult time discerning legitimate journals from those that exploit the world of publishing, making the *Canadian Journal of Pain* a logical and trustworthy choice.

I am confident that within a few short years the *Canadian Journal of Pain* will be a trusted and highly-respected outlet for innovative ideas and empirical research in the field of pain. We look forward to receiving your manuscripts and to working with you as we strive for a painless publishing experience.

